# Infestation of parasitic rhizocephalan barnacles *Sacculina beauforti* (Cirripedia, Rhizocephala) in edible mud crab, *Scylla olivacea*

**DOI:** 10.7717/peerj.3419

**Published:** 2017-06-30

**Authors:** Khor Waiho, Hanafiah Fazhan, Henrik Glenner, Mhd Ikhwanuddin

**Affiliations:** 1 Institute of Tropical Aquaculture, Universiti Malaysia Terengganu, Kuala Terengganu, Terengganu, Malaysia; 2 Marine Biology Institute (MBI), Shantou University, Shantou, Guangdong, China; 3 Marine Biodiversity Group, Department of Biology, University of Bergen, Bergen, Norway; 4 Center for Macroecology and Evolution, University of Copenhagen, Copenhagen, Denmark

**Keywords:** Mud crab, *Sacculina beauforti*, Rhizocephalan, *Scylla olivacea*, Parasites, Sacculinids, Mitochondrial COI

## Abstract

Screening of mud crab genus *Scylla* was conducted in four locations (Marudu Bay, Lundu, Taiping, Setiu) representing Malaysia. *Scylla olivacea* with abnormal primary and secondary sexual characters were prevalent (approximately 42.27% of the local screened *S. olivacea* population) in Marudu Bay, Sabah. A total of six different types of abnormalities were described. Crabs with type 1 and type 3 were immature males, type 2 and type 4 were mature males, type 5 were immature females and type 6 were mature females. The abdomen of all crabs with abnormalities were dented on both sides along the abdomen’s middle line. Abnormal crabs showed significant variation in their size, weight, abdomen width and/or gonopod or pleopod length compared to normal individuals. The mean body weight of abnormal crabs (type 1–5) were higher than normal crabs with smaller body size, while females with type 6 abnormality were always heavier than the normal counterparts at any given size. Sacculinid’s externa were observed in the abdomen of crabs with type 4 and type 6 abnormalities. The presence of embryos within the externa and subsequent molecular analysis of partial mitochondrial COI region confirmed the rhizocephalan parasite as *Sacculina beauforti*. Future in-depth descriptions of the life cycle and characteristics of *S. beauforti* are recommended as it involves a commercially important edible crab species and the effect on human health from the consumption of crabs is of crucial concern.

## Introduction

Alteration in the morphological primary and secondary sexual characteristics in crabs are possible and does occur in nature (Høeg, 1984; Rees & Glenner, 2014). One of the most common causes reported are specific parasitic infection (Ayaki et al., 2005). The resulting effects such as alteration of the sexual characters and disruption of normal gonad maturation and copulation processes are often permanent and adversely affect the host’s population and local biodiversity (Ruiz et al., 1999).

It is well-known that rhizocephalan parasites cause sterilisation and feminisation of their hosts (Ayaki et al., 2005). The most notable characteristic of rhizocephalan infections is the emergence of a yellow sac-like structure, known as externa within the external abdomen cavity of its host. The externa contains the reproductive organs of the female parasite (Rees & Glenner, 2014). The parasite feeds on the nutrient extracted from the hemolymph of its host via a root-like structure known as the interna (Glenner, 2001). Infected crabs are unable to moult after the formation of externa (Høeg, 1984; Piper, 2007). Once a virgin female externa is fertilized by a dwarf male, the infected crab will look after the fertilised eggs of the invaders until they hatch. Rhizocephalan parasites, including sacculinids, are known to infect both sexes of crabs, and upon infection sterilising all their hosts. The infected male will be feminised and shows morphological and behavioural alterations, typical in females, such as widening of the abdomen, reduction in chela size, and typical egg caring and releasing behaviour (Boschma, 1955; Høeg, 1984; Piper, 2007).

The prevalence of parasitic barnacles is reported around the world, including European countries (Rees & Glenner, 2014), Japan (Lutzen & Takahashi, 1997), Taiwan (Huang & Lutzen, 1998), China (Yang et al., 2014), Malaysia (Boschma, 1949), Australia (Knuckey, Davie & Cannon, 1995), America (Tolley et al., 2006) and India (Raffi et al., 2012). As well as mud crabs, rhizocephalan parasites are known to invade a wide range of other marine and intertidal crab species such as the European green crab, *Carcinus maenas* (LINNAEUS 1758) (Thresher et al., 2000), intertidal crab, *Hemigrapsus sanguineus* (DE HAAN 1853) (Lutzen & Takahashi, 1997), sand crab, *Portunus pelagicus* (LINNAEUS 1758) (Bishop & Cannon, 1979; Weng, 1987) and three-spotted crab, *Portunus sanguinolentus* (HERBST 1783) (Raffi et al., 2012; Yang et al., 2014).

Targeting mostly brachyuran crabs, this parasitic infestation, together with the effect of overexploitation, threaten the fishery industry on a global scale as most economically important crab species are brachyuran crabs from the family Portunidae (Raffi et al., 2012). The sterilisation effect of parasites resulting from the alteration of morphological characters and hormonal levels (Rubiliani, 1983) reduce overall reproduction rates, therefore lowering population densities over time. If left unattended, it may ultimately end with the extinction of the host species in the infected population. Two species of rhizocephalan parasites were reported to parasitize mud crab genus *Scylla*, i.e., *Loxothylacus ihlei* BOSCHMA 1949 and *Sacculina beauforti* BOSCHMA 1949, with the latter being reported in Sandakan Bay, Sabah (Boschma, 1949; Knuckey, Davie & Cannon, 1995).

From the year 2008 to 2015, the quantity and value of Malaysia’s inshore fisheries were consistently higher than that of deep sea fisheries. The landing of inshore fisheries in 2015 was more than 1,100,000 tonnes and worth approximately RM8,000 million. Together with other smaller fisheries landing species such as bivalves and sea crabs, mud crabs showed the highest landings in the east coast of peninsular Malaysia, west coast of peninsular Malaysia, and East Malaysia, with landing values of approximately 160,000, 2,005,000 and 200,000 tonnes respectively (Department of Fisheries Malaysia, 2016). Traditional crab pots are commonly used by local fishermen to capture mud crabs along mangrove forests, river and estuarine coastlines, and intertidal flats. Being considered as one of the important fishery commodities in Malaysia, mud crabs are highly sought after, both locally and internationally (Fazhan et al., 2017; Waiho et al., 2015; Waiho, Fazhan & Ikhwanuddin, 2016).

A recent preliminary survey revealed that some forms of abnormalities were present in the mud crab population of Marudu Bay, Sabah, Malaysia, during sampling for previous works of Waiho, Fazhan & Ikhwanuddin (2016) and Fazhan et al. (2017). In addition, we observed that locals prefer abnormal crabs due to their fullness in meat based on their higher body weight (BW) compared to the normal mature mud crabs. Interviews with the local crab sellers revealed that this phenomenon has been prevalent for more than five years. This is unusual as most hosts infested by rhizocephalan parasites exhibit reduced growth rates (O’Brien & Van Wyk, 1985) and decreased in feeding behaviour (Belgrad & Griffen, 2015). Therefore, the objectives of the present study were to verify the infestation of rhizocephalan parasite in the mud crab genus *Scylla,* to characterize the different types of abnormalities present in the *Scylla* population and to compare their length (carapace width (CW)), weight (BW) and secondary sexual related organs (abdomen width (AW), gonopod length (GL), pleopod length (PL)) with that of the normal crabs, in order to assess the impact of this parasite towards the *S. olivacea* population.

## Materials & Methods

### Screening

Screening of the mud crab genus *Scylla* from four locations in Malaysia (i.e., Marudu Bay-Sabah (6°44′N117°1′E), Lundu - Sarawak (1°40′N109°58′E), Taiping - Perak (4°45′N100°37′E) and Setiu - Terengganu (5°39′N102°43′E)) was conducted from June 2012 to July 2013. Additional mud crab screening at Marudu Bay, Sabah was carried out until December 2013. These four locations were chosen based on their high mud crab landings (Fazhan et al., 2017; Waiho, Fazhan & Ikhwanuddin, 2016). Being one of the economically important coastal natural resources, mud crabs are harvested from all four locations consistently by local fishermen to meet the demand of domestic and international markets. Thus, no specific permission was required for the sampling of mud crabs in these common fishing grounds. Mud crab species identification was based on the morphological keys described by Keenan, Davie & Mann (1998). Preliminary screening revealed that out of the three species available, only *S. olivacea* from Marudu Bay, Sabah showed visible abnormalities of the sexual organs. Therefore, data of *S. tranquebarica* and *S. paramamosain* were excluded from this study. The quantity, CW, BW and AW (measurement of the greatest width of the 5th segment of abdomen segment) of *S. olivacea* from all four sampling locations were recorded. Crabs with missing or partially regenerated appendages were not included in this study.

### Classification of abnormal crabs

Crabs with abnormalities were classed based on their differences and their deformities described. Maturity status was determined by inspecting the internal gonadal development of both sexes. Crabs were anesthetised (immersion in ice water for 5 min) and then sacrificed (destruction of the dorsal ganglia). In addition, characters i.e., male’s GL, female’s PL and externa diameter (ED) were measured to the nearest 0.01 mm using a standard Vernier calliper. Female’s PL referred to the length of the first pair of pleopod near the base of the abdomen, while the ED was the largest diameter of the externa.

### Length-weight relationship

The length-weight relationship of the crab can be described by the formula: *W* = *aL*^*b*^, where *W* = BW(g), *L* = length (or CW in this case) (mm), *a* = constant and *b* = regression slope (Hile, 1936). The BW and CW of crabs were log-transformed and plotted on scatter diagrams to determine the length-weight relationships using linear regression analysis. The formula after logarithmic transformation is: log BW = log *a* + *b* log CW.

### Microscopic and molecular identification of parasite

Microscopic examination of the externa was conducted. The externa was removed and observed under a stereoscopic zoom microscope (LEICA EZ4). Further confirmation of rhizocephalan was conducted by molecular analysis. DNA was extracted from the externa using the GF-1 Nucleic Acid DNA extraction kit (Vivantis Technologies, Selangor, Malaysia). The Cytochrome Oxidase 1 (COI) gene was amplified using universal primer LCO1490 and HCO 2198 (Folmer et al., 1994; Fazhan, Waiho & Shahreza, 2016). The polymerase chain reaction (PCR) was carried out with 35 cycles of 25 μl reaction volume containing 2.5 μl of 1 × PCR buffer (Biotaq, Gaithersburg, MD, USA), 1 μl dNTP (2.5 mM), 1 μl of each primer (10 μM), 0.5 μl taq polymerase (Biotaq, Gaithersburg, MD, USA), and 2 μl of DNA template. The temperature profile was: 94°C for 10 min (predenaturation), followed by 35 cycles of denaturation at 94°C for 30 s, annealing at 52°C for 30 s, extension at 72°C for 45 s, and a final extension of 72°C for 10 min. PCR products were then sent for sequencing. Early and end signal losses of the DNA sequences were trimmed prior to further analysis. The partial COI sequence was aligned with the National Center of Biotechnology Information (NCBI) BlastN database by using BLAST (Basic Local Alignment Search Tool) algorithm for possible species identification (Altschul et al., 1997). The COI sequence of *Sacculina beauforti* obtained in this study, and 18 additionally selected rhizocephalan sequences downloaded from GenBank, was compiled into a single Fasta file. The sequences were subsequently imported into SeaView (SeaView, version 4.5.4, 2015) and aligned using the Clustal Omega software (Clustal Omega, version 1.2, 2015) built into the application. A parsimony analysis of the aligned rhizocephalan COI sequences was performed in the Seaview program package, where character states were unordered, weighted equally, and alignment gaps were coded as missing data. The most parsimonous topology was evaluated by bootstrap resampling (Swofford, 1998) for 500 replicates, each with 25 random-addition heuristic searches.

**Table 1:** The maximum value (Max), minimum value (Min) and average value (Mean) in terms of CW, BW, AW, GL, PL and ED of *Scylla olivacea* (normal crabs and crabs with abnormalities—type 1–6) from Marudu Bay (Sabah), Lundu (Sarawak), Taiping (Perak) and Setiu (Terengganu).

		Marudu Bay, Sabah									
		IM	MM	IF	MF	Type 1	Type 2	Type 3	Type 4	Type 5	Type 6
CW (mm)	Max	103.98	126.11	105.56	119.9	95.84	121.41	109.52	141.32	103.41	120.96
	Min	64.7	74.18	62.45	80.28	67.39	83.57	68.07	86.94	73.87	87.71
	Mean	83.28 ± 8.11	98.31 ± 10.52	83.61 ± 9.36	100.46 ± 8.92	81.42 ± 6.84	100.77 ± 10.51	82.69 ± 10.32	106.74 ± 11.66	91.49 ± 7.58	105.19 ± 8.83
BW (g)	Max	213.2	520.4	189.8	319.7	200.3	477.2	260.8	688.2	186.2	286.6
	Min	46.0	80.8	44.1	89.1	49.1	100.3	61.1	161.5	75.9	117.5
	Mean	115.30 ± 35.17	206.92 ± 92.56	104.20 ± 28.99	171.25 ± 42.33	105.99 ± 29.45	216.16 ± 89.82	131.2 ± 46.48	266.59 ± 104.16	128.37 ± 28.16	203.45 ± 48.62
AW (mm)	Max	19.77	26.8	33.64	52.47	25.98	36.19	24.88	43.26	32.46	45.21
	Min	12.99	15.86	14.41	24.4	14.33	21.33	14.55	24.06	20.93	29.96
	Mean	16.36 ± 1.50	19.72 ± 2.16	24.46 ± 3.39	38.22 ± 6.42	17.82 ± 1.82	27.96 ± 4.01	18.62 ± 2.52	31.07 ± 4.12	26.19 ± 2.73	39.49 ± 3.59
GL (mm)	Max	22.13	26.48	–	–	19.83	26.34	7.48	8.43	–	–
	Min	12.80	15.62	–	–	12.8	15.94	3.61	3.8	–	–
	Mean	17.46 ± 1.92	20.65 ± 2.40	–	–	16.34 ± 1.57	20.89 ± 2.25	5.27 ± 0.89	6.22 ± 1.22	–	–
PL (mm)	Max	–	–	36.02	39.82	–	–	–	–	7.61	7.26
	Min	–	–	20.39	26.5	–	–	–	–	2.72	3.32
	Mean	–	–	26.78 ± 3.81	33.08 ± 3.15	–	–	–	–	4.80 ± 1.16	5.15 ± 0.95
ED (mm)	Max	–	–	–	–	–	–	–	31.23	–	31.68
	Min	–	–	–	–	–	–	–	18.46	–	18.03
	Mean	–	–	–	–	–	–	–	23.68 ± 3.00	–	24.63 ± 3.10
*n*		163	179	136	157	93	87	79	49	63	94
*N*		1,100									

		Lundu, Sarawak				Taiping, Perak				**Setiu, Terengganu**			
		IM	MM	IF	MF	IM	MM	IF	MF	IM	MM	IF	MF
CW (mm)	Max	90.39	134.42	98.25	114.83	92.75	121.98	91.92	124.90	91.05	106.96	91.21	110.57
	Min	67.09	80.05	65.59	82.30	72.76	77.96	69.51	81.50	56.89	69.61	61.02	71.38
	Mean	79.11 ± 5.82	100.16 ± 9.75	79.03 ± 6.96	101.04 ± 8.01	79.85 ± 5.52	95.15 ± 7.91	78.36 ± 4.67	98.33 ± 8.15	75.64 ± 6.91	87.06 ± 8.14	75.63 ± 7.62	90.71 ± 8.80
BW (g)	Max	162.2	719.2	193.9	268.4	172.9	444.8	133.2	308.1	161.9	345.0	140.0	217.0
	Min	50.4	102.3	50.5	85.0	72.5	96.9	63.2	94.2	35.0	62.9	43.3	70.5
	Mean	95.13 ± 24.23	237.62 ± 101.88	92.59 ± 27.52	177.54 ± 38.80	100.75 ± 25.99	190.89 ± 67.45	87.83 ± 14.08	155.31 ± 40.76	92.62 ± 26.46	147.38 ± 49.91	84.35 ± 23.80	132.6 ± 29.05
AW (mm)	Max	18.24	28.96	32.83	53.54	18.79	23.68	36.16	54.22	18.47	24.05	35.71	49.74
	Min	12.89	17.12	17.35	23.06	12.98	15.21	19.90	32.09	10.48	14.48	13.52	14.29
	Mean	15.77 ± 1.20	20.38 ± 2.48	24.02 ± 3.53	41.30 ± 7.17	15.11 ± 1.34	19.13 ± 1.56	24.83 ± 2.66	42.38 ± 4.44	14.99 ± 1.58	18.17 ± 1.74	24.03 ± 3.70	34.76 ± 8.23
GL (mm)	Max	18.76	27.91	–	–	18.41	25.93	–	–	18.05	23.29	–	–
	Min	12.80	16.04	–	–	13.74	16.13	–	–	10.74	14.00	–	–
	Mean	15.79 ± 1.21	21.33 ± 2.07	–	–	15.71 ± 1.15	19.92 ± 1.94	–	–	14.79 ± 1.49	18.58 ± 1.83	–	–
PL (mm)	Max	–	–	30.14	40.66	–	–	28.07	39.85	–	–	28.64	38.66
	Min	–	–	19.41	25.61	–	–	19.29	24.60	–	–	17.03	22.31
	Mean	–	–	23.47 ± 2.33	32.86 ± 3.35	–	–	22.94 ± 1.78	32.10 ± 3.42	–	–	22.28 ± 2.55	29.67 ± 3.48
ED (mm)	Max	–	–	–	–	–	–	–	–	–	–	–	–
	Min	–	–	–	–	–	–	–	–	–	–	–	–
	Mean	–	–	–	–	–	–	–	–	–	–	–	–
	*n*	108	134	94	116	88	101	90	133	121	75	91	80
	*N*	452				412				367			

**Notes.**

IM immature male MM mature male IF immature female MF mature female CW carapace width BW body weight AW abdomen width GL gonopod length PL pleopod length ED externa diameter

### Data analysis

Statistical analyses were conducted using Microsoft Excel 2013 and IBM SPSS version 20. All data were checked for normality (Shapiro–Wilk test) and homogeneity of variance (Levene’s Test) prior subjecting to standard parametric tests. Data of normal crabs (*S. olivacea*) from all four locations were pooled and compared with crabs with abnormalities. Analysis of variance (ANOVA) with welch correction was used to determine if there was any significant difference in CW, BW, AW, GL and PL based on abnormality. Post hoc Games Howell test was used to compare between treatments if differences were significant and a standard independent *t*-test was used to compare the means of ED. Analysis of covariance (ANCOVA) was used to compare between regression lines to determine the length-weight relationships of normal and abnormal crabs. All tests assumed significant level at 5%. Data are presented as mean ± SD (standard deviation).

## Results

### Classification of abnormal crabs

**Figure 1:**
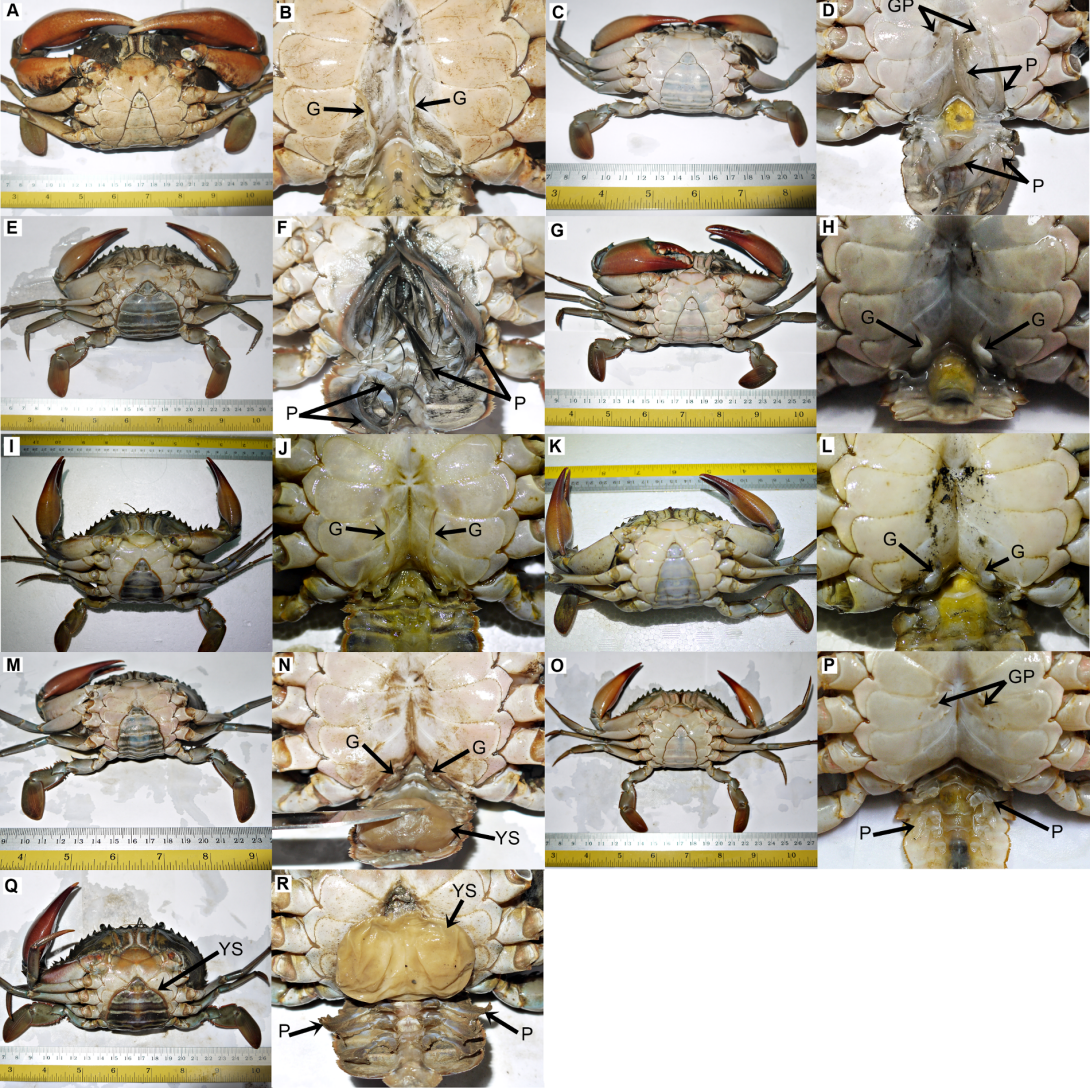
The ventral view of normal and abnormal *Scylla olivacea.* The gonopods (G), gonopores (GP), pleopods (P) and externa (YS) are labelled with arrows. (A and B) normal male; (C and D) normal immature female; (E and F) normal mature female; (G and H) crab with type 1 abnormality; (I and J) crab with type 2 abnormality; (K and L) crab with type 3 abnormality; (M and N) crab with type 4 abnormality; (O and P) crab with type 5 abnormality; (Q and R) crab with type 6 abnormality.

A total of 2,341 crabs were examined throughout the study period. Parasitism of rhizocephalan was found in both sexes of *Scylla olivacea*, with a high occurrence of 42.27% (465 out of 1,100 crabs) in Marudu Bay, Sabah, while no abnormal crab was sighted in the other three locations (Table 1). The detailed abnormalities were described and categorised as in Table 2 and Fig. 1. The occurrence of crabs with type 1–6 abnormalities compared with the population in Marudu Bay, Sabah were 6.36%, 6.85%, 6.36%, 3.42%, 6.36% and 9.30% respectively. All abnormalities occurred at the abdomen area and affected the pleopods in females or gonopods in males. The abdomen segment was dented at both sides along the middle line, an uncommon characteristic that was only shared in all abnormal crabs (Figs. 1G–1R). In addition, the presence of a yellow sac (externa) was observed in crabs with type 4 (male) (Fig. 1N) and type 6 (female) (Fig. 1R) abnormalities. All crabs with abnormalities type 2, 4 and 6 were found to be mature crabs based on the presence of enlarged milky white vas deferens in males, and darkened and widened abdomen in females. From macroscopic examination, it was observed that all examined type 6 females exhibited ovarian maturation status of stage II–IV, but with an obvious reduction in ovarian mass compared to normal mature females.

**Table 2:** Description of abnormalities in males and females of *Scylla olivacea.*

Abnormality	Characteristics						
		Abdomen coloration	Abdomen shape	Gonopods length	Pleopods length	Dented abdomen	Presence of externa	Maturity status
Male	Type 1	Normal	Slightly globular	Normal	–	Yes	No	Immature
	Type 2	Darkened	Widened and globular, female-like	Normal	–	Yes	No	Mature
	Type 3	Normal	Slightly globular	Reduced	–	Yes	No	Immature
	Type 4	Darkened	Widened and globular, female-like	Reduced	–	Yes	Yes	Mature
Female	Type 5	Normal	Slightly globular (as in normal immature females)	–	Reduced	Yes	No	Immature
	Type 6	Darkened	Widened and globular (as in normal mature females)	–	Reduced	Yes	Yes	Mature

### Morphometric characteristics of normal and abnormal crabs

The CW (*Welch’s F*(9, 394.54) = 294.64, *P* < 0.001), BW (*Welch’s F*(9, 390.83) = 216.93, *P* < 0.001) and AW (*Welch’s F*(9, 388.53) = 1303.59, *P* < 0.001) were significantly different between crabs of different abnormality status and types. Subsequent post hoc test revealed that there was no significant difference between the CW of male crabs with type 1 abnormality with normal immature males (*P* = 0.547) and type 3 males (*P* = 0.995). The CW of males with type 2 abnormality was not significantly different with that of males with type 4 abnormality (*P* = 0.102) and both were significantly larger in size (CW) compared to mature males (*P*_type 2_ < 0.019; *P*_type 4_ < 0.001). The CW of females with type 5 abnormality was greater than that of immature females (*P* < 0.001) but still smaller than the CW of normal mature females (*P* < 0.001). The CW of females with type 6 abnormality was larger than the CW of mature females (*P* < 0.001), but not significantly different to crabs with type 2 and type 4 abnormalities (*P*_type 2_ = 0.077; *P*_type 4_ = 0.998).

The BW did not follow the same pattern as CW. The BW of males with type 1 abnormality was significantly smaller (all *P* < 0.002) compared to the BW of other crab types, except with that of immature males (*P* = 0.986). The BW of males with type 4 abnormality was significantly higher than the BW of other crab types (all *P* < 0.018) and not significantly different with the BW of males with type 2 abnormality (*P* = 0.138). The BW of males with type 2 abnormality was similar to that of mature males (*P* = 0.958), but significantly heavier than all the remaining crab types (all *P* < 0.001). The BW of crabs with type 3 and type 5 abnormalities showed a similar pattern, i.e., no significant difference between them (*P* = 1.000), significantly heavier than normal immature males and immature females (all *P* < 0.001) but lighter than normal mature males and mature females (all *P* < 0.001). Females with type 6 abnormality however, were significantly heavier than normal mature females (*P* < 0.001).

Although no significant difference was found between the AW of males with type 1 and type 3 abnormalities (*P* = 0.0376), they were larger than that of normal immature males (*P* < 0.001). The AW of males with type 2 and type 4 abnormalities were considerably larger than the AW of normal immature and mature males (all *P* < 0.001). When a comparison was made between them, the AW of males with type 4 abnormality was significantly larger than the AW of males with type 2 abnormality. Females with type 5 and type 6 abnormalities showed a similar pattern as well, i.e., wider AW compared to that of normal immature females (both *P* < 0.001). Interestingly, none of the crabs with abnormalities exhibited AW wider than the AW of normal mature females (all *P* < 0.001).

The GL between normal males and males with type 1–4 abnormalities were significantly different (*Welch’s F*(5, 236.82) = 2533.85, *P* < 0.001). In normal condition, mature males exhibited significantly longer GL compared to immature males (*P* < 0.001). The GL of males with type 1 and type 4 abnormalities were comparable to that of normal immature males (*P*_type 1_ = 0.759; *P*_type 4_ = 0.083), whereas the GL of males with type 2 abnormality was similar to that of normal mature males (*P* = 0.360). The GL of males with type 3 abnormality was the shortest among all types of abnormal and normal males (all *P* < 0.001).

For PL, significant differences were observed between different groups (i.e., females of type 5 and type 6 abnormalities, and normal immature and mature females) (*Welch’s F*(3, 305.62) = 9415.26, *P* < 0.001). Subsequent post hoc test revealed that the mean PL was similar between females with type 5 and type 6 abnormalities (*P* = 0.199), but was significantly shorter (both *P* < 0.001) compared to the PL of normal females regardless of maturity status (${\bar {x}}_{\mathrm{type}~5} \text{{\XMLAMP}} {\bar {x}}_{\mathrm{type}~6}\lt {\bar {x}}_{\mathrm{immature~ female}}\lt {\bar {x}}_{\mathrm{mature~ female}}$).

In addition, the ED ranged from 18.03 mm to 31.68 mm. No significant difference (*t*(141) = 1.775, *P* = 0.078) in ED was found between crabs infected with rhizocephalan parasites, i.e., males with type 4 abnormality and females with type 6 abnormality.

Thus, crabs with type 1, 3 and 5 abnormalities were the immature crabs of crabs with type 2, 4 and 6 abnormalities, respectively based on their deformed characters (Table 2), maturity status and the differences in body size and weight.

### Length-weight relationships of normal and abnormal crabs

**Figure 2:**
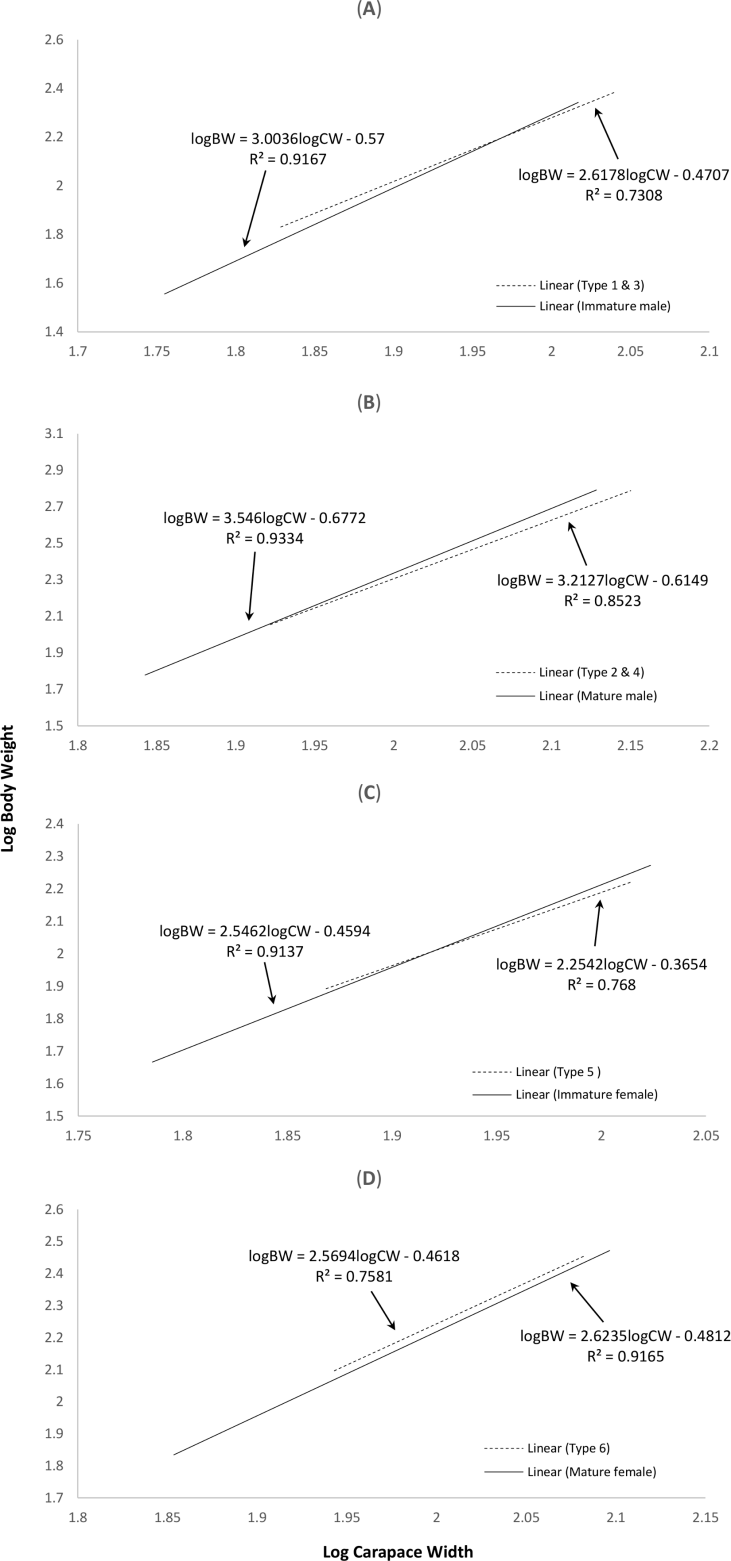
Log-transformed length-weight relationships of (A) normal immature males and crabs with type 1 & 3 abnormalities, (B) normal mature males and crabs with type 2 & 4 abnormalities, (C) normal immature females and crabs with type 5 abnormality, and (D) normal mature females and crabs with type 6 abnormality. The interception points in terms of carapace width in (A), (B) and (C) are 93.76 mm, 80.83 mm and 82.95 mm, respectively.

The correlation between weight and length of the normal *S. olivacea* was stronger compared to that of abnormal individuals, as indicated by their higher correlation coefficient values (*r* = 0.95 − 0.97 for normal crabs; *r* = 0.85 − 0.92 in abnormal crabs). The regression slope *b* values were consistently lower in abnormal crabs compared to normal crabs (Fig. 2). The abnormal crabs (type 1 & 3, type 2 & 4, and type 5) were heavier at smaller CW and after reaching the intercepts of the two linear regression lines, the normal crabs had a higher mean BW (Figs. 2A–2C). The linear regression line of crabs with type 6 abnormality were on top of and almost parallel to the regression line of mature females (Fig. 2D), indicating that at any given size, the crabs with type 6 abnormality were heavier than the normal mature females.

### Identification of *Sacculina beauforti*

**Figure 3:**
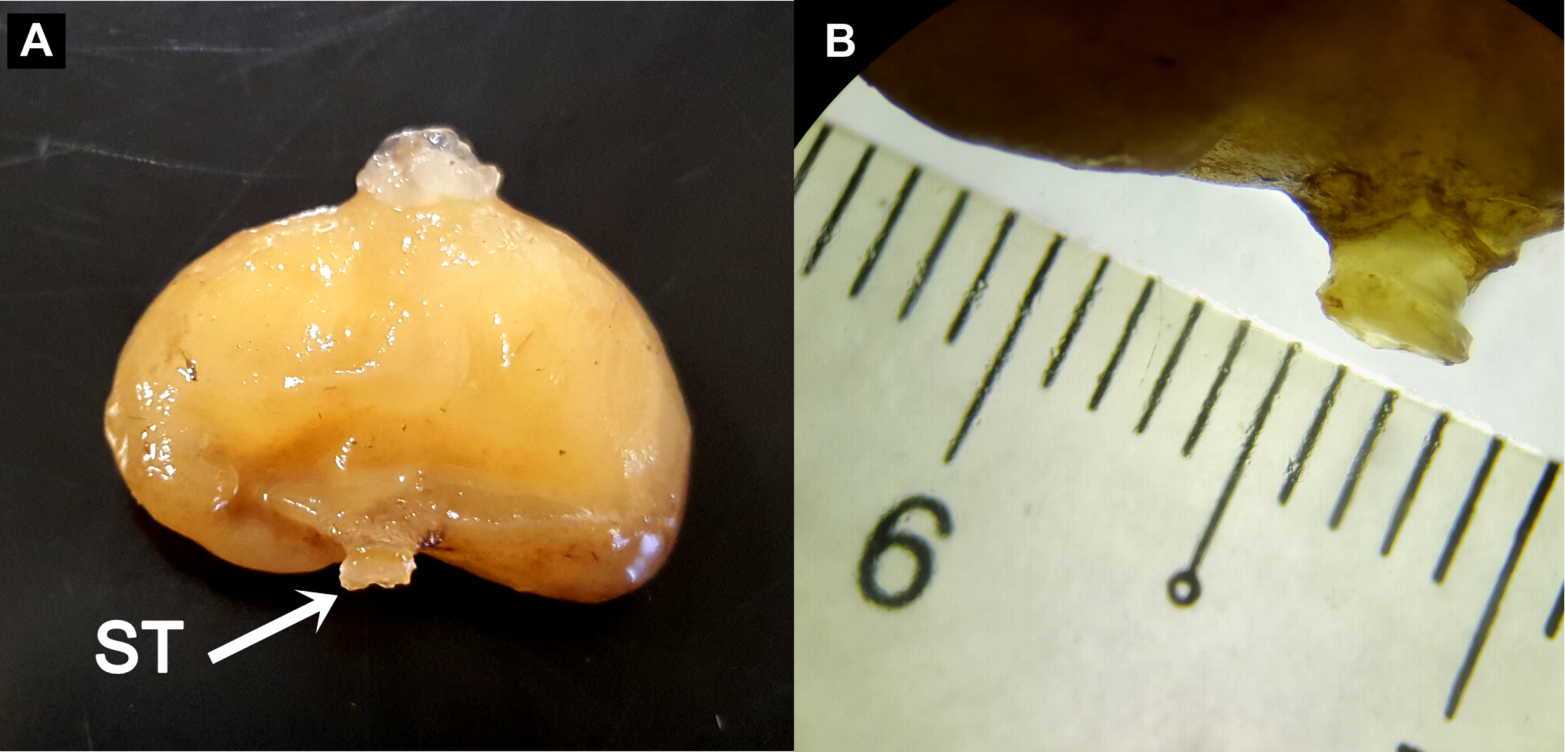
The ovoid-shaped externa of rhizocephalan parasite. (A) the external morphology of rhizocephalan’s externa, with its stalk (ST) being pointed out with arrow. (B) Close-up of externa’s stalk (approximately 3 mm in diameter) viewed under stereoscopic zoom microscope.

**Figure 4:**
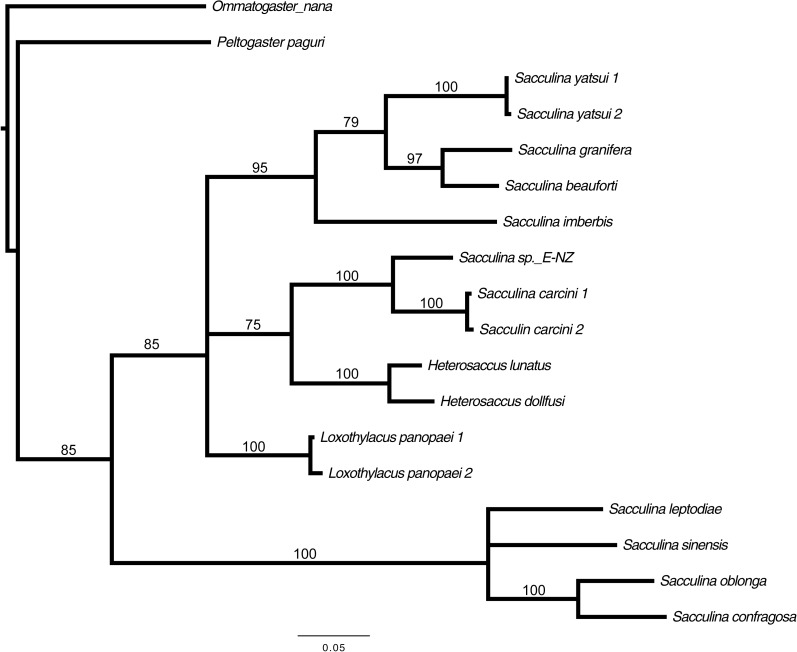
Strict consensus of 3 most parsimonious trees, 691 sites (580 informative), gaps treated as missing data. Bootstrap support values from 500 replicates are shown below each branch. Values below 50 are not indicated. Two rhizocephalans (*Ommatogaster nana* and *Peltogaster paguri*) not from the family Sacculinidae were used as outgroup.

Preliminary assessment of the externa revealed that the outer envelope of the externa was quite thick and strong (able to withstand normal finger’s press pressure). The externa was found to be attached at the base of the abdomen segment, located in-between the first and second pairs of pleopods in females or after the gonopods in males. Being relatively large in terms of size and dimension, it occupied the entire abdomen and caused the abdomen segment to be always protruded. The externa’s stalk was apparent upon removal of the externa from the infected crab’s abdomen (Fig. 3). Based on the description of externa and its attachment site at the abdomen of infected crabs, it is confirmed that crabs with type 4 and type 6 abnormalities were infected with rhizocephalan parasites, *Sacculina beauforti*. This was further validated by the results of BLAST analysis. When compared to the online NCBI nucleotide database, the PCR products (612 bp) of externa obtained in the current study (deposited into GenBank with the accession number KX426583) showed highest (maximum identity percentage = 91%) similarity with the partial COI gene of a rhizocephalan, *Sacculina granifera* BOSCHMA 1973 (Sacculinidae) (GenBank accession number = DQ059779.1) (query cover = 100%, *E*-value = 0.0). The parsimonious tree (Fig. 4) places *S. beauforti* close to *S. granifera* in a clade of East Asian sacculinids.

## Discussion

The body size and weight of crabs with abnormalities were larger than their normal counterparts, i.e., immature and mature males and females. The lower regression slope *b* values in abnormal *S. olivacea* were also reported in the swimming crab *P. sanguinolentus* infected with thompsoniid *Diplothylacus sinensis* (KEPPEN 1877) (Yang et al., 2014). The higher body weight of crabs with abnormalities (infected) with a smaller body size found in this study is consistent with the preliminary observations and comments by local crab sellers, in which the abnormal crabs were preferred over normal mature males of the same size, by the locals, for consumption due to their heavier body weight. This phenomenon is not common in crabs infected with rhizocephalan parasites. Parasitized crabs are generally smaller in size and weighed significantly less than non-parasitized individuals (Hawkes, Meyer & Shirley, 1986; O’Brien & Van Wyk, 1985; Yang et al., 2014), indicating that the attached parasites were depleting the host’s energy reserves (Belgrad & Griffen, 2015). However, different rhizocephalan species might exert different effects on their hosts, and even the same parasite might have varying damaging effects to different hosts (Li et al., 2011; Okada & Miyashita, 1935; Yang et al., 2014). To this point, we are still trying to understand the full effects of *S. beauforti* infestation on the growth, reproduction and defence mechanism of *S. olivacea*. Belgrad & Griffen (2015) demonstrated that infected crabs showed no difference in metabolism and digestion times with that of uninfected crabs, but were less active, with an almost five-fold decrease in feeding behaviour and 20% increase in hiding behaviour. They postulated that although the increase in hiding behaviour means a decrease in foraging time, which could cause starvation and potentially affect a crab’s survival (Toscano, Newsome & Griffen, 2014), it is balanced off by the decrease in metabolic demands when crabs spend more time inactive. In a separate experiment conducted by Larsen, Høeg & Mouritsen (2013) on the effect of *Sacculina carcini* THOMPSON 1836 infection on the consumption rate in crab *C. maenas*, it was found that parasitic infection of *S. carcini* had a limited effect on the feeding biology of its hosts. If *S. beauforti* caused similar behavioural changes in *S. olivacea*—increase in hiding behaviour and/or maintain their metabolism, digestion and feeding biology, and coupled with the inability to molt after being parasitized, the food intake of the host will be converted into more muscle mass and flesh. This might explain why infected *S. olivacea* were heavier than uninfected individuals and thus much preferable by the locals. Future studies should therefore explore the behavioural differences in infected and uninfected *S. olivacea*, especially their foraging and feeding behaviours, in attempt to explain the unusual phenomenon found in our study.

In general, sexual related organs (AW, GL and PL) of infected crabs were greatly altered, and the number of infected crabs was high (i.e., more than a quarter of the normal populations). The modification of the secondary sexual related organs of host crabs after rhizocephalan parasite infestation is expected (Knuckey, Davie & Cannon, 1995; Yang et al., 2014). The occurrence of *S. beauforti* externae in *S. olivacea* was high (12.88% throughout the Sabah *S. olivacea* population). In addition, based on the abdomen shape and coloration, it is almost certain that crabs with type 1, 2, 3 and 5 abnormalities were crabs that already host an internal *S. beauforti*, but the externa has yet to emerge to the exterior of the abdomen of the crab, as crabs parasitized by rhizocephalan parasites are feminized gradually via molts. It is only when the externa emerges to the exterior that the molting circle of the host is arrested (Rubiliani, 1983; Glenner, 2001). The feminisation of male hosts and change in the sexual related organs of *S. olivacea* by *S. beauforti* were apparent, although not all hosts manifest externae. This triggered a hypothetical question: are crabs with abnormalities fertile and able to reproduce? The large number and the presence of mature gonads in both sexes indicate that they might be able to reproduce normally. However, the reduction in length of gonopods in abnormal males imply that successful copulation is very unlikely to occur as the reduced gonopods are clearly too short to enter the gonopores of females, thus preventing the transfer of spermatophores. Even if copulation successfully occurred between a normal male and abnormal female, the copulated female is unable to secure her fertilized eggs onto her reduced pleopods. In addition, the sterilisation effect onto infected hosts by rhizocephalan parasites are well documented (Isaeva, Dolganov & Shukalyuk, 2005; Noever, Olson & Glenner, 2016; Yang et al., 2014), even in sacculinids (Høeg, 1995). Further studies on the histology and mating of abnormal crabs are recommended to validate these hypotheses.

The identity of the rhizocephalan parasite infecting *S. olivacea* was confirmed to be *Sacculina beauforti* with the high sequence similarity of *S. beauforti* partial COI gene sequences with that of other Sacculinids found in GenBank. This is the second report of the occurrence of *S. beauforti* on the mud crab genus *Scylla* in Borneo, in which the first occurrence was reported more than six decades ago (Boschma, 1949), thus justifying the lack of *S. beauforti* DNA sequence in any publicly available DNA databases. Previously, *S. beauforti* was reported on the mud crab *S. serrata* (Boschma, 1949). However, a recent revision of genus *Scylla* by Keenan, Davie & Mann (1998) divided this once single-species genus into four distinct species (*S. serrata, S. olivacea, S. tranquebarica* and *S. paramamosain*). Thus, it is most likely that the mud crab species infected with *S. beauforti* described by Boschma (1949) was *S. olivacea*, similar to the findings in this study as no *S. serrata* was reported in Malaysian waters after Keenan’s revision.

There is limited information on the host specificity of rhizocephalan parasites and while some rhizocephalan species are fairly specific in choosing their hosts, others were reported to exhibit a broad host compatibility (Boone et al., 2004; Poltev, 2008). For example, the rhizocephalan parasite *Loxothylacus texanus* (BOSCHMA 1933) were found on crabs of different families, including grapsid *Sesarma cinereum* (BOSC 1802), xanthid *Rhithropanopeus harrisii* (GOULD 1841) and portunid *Callinectes* spp. STIMPSON 1860 (Boone et al., 2004). However, it was postulated that physical and biological factors such as the successful penetration of the vermigon into the hemolymph of the host and the evasion of interna from the host’s immune responses might be pivotal in determining the success of infecting a new host by a rhizocephalan parasite (Boone et al., 2004). An experiment conducted on the host specificity of *S. carcini* THOMPSON 1836 to infect its natural host, the European green crab *C. maenas*, and four other non-target California crabs showed that the cyprid larvae of *S. carcini* settled in higher numbers on its natural host and unlike *C. maenas*, all infected native California crabs died without exhibiting an externa (Goddard et al., 2005). The sacculinids *S. beauforti* were only found on *S. olivacea* and absent in the other two *Scylla* species in this study. This suggests that although host specificity of *S. beauforti* could yet be proven, we could assume that its natural host is *S. olivacea*. Possible infections of *S. beauforti* in *S. paramamosain* and *S. tranquebarica* might occur occasionally (at a lower number) in the wild but due to the specificity needed in the composition, amount and timing to release controlling substances by the rhizocephalan to affect the nervous and endocrine systems of its host (Høeg, 1995), the non-host *Scylla* species are more likely to die without developing externa, thus avoiding detection during our screening. It is also reported that some crab species are more resistant to infection and the effects of parasitism than others (Hawkes, Meyer & Shirley, 1986). In addition, physical parameters such as water salinity affects the prevalence of rhizocephalan parasites (Tolley et al., 2006). Tolley et al. (2006) found that rhizocephalans *Loxothylacus panopaei* (GISSLER 1884) were absent upstream during wet months and estuaries with high freshwater inflow. Positive correlation between the parasite prevalence and the mean salinity of host crabs were also reported. Therefore, the prevalence of *S. beauforti* might be confined to certain physical parameters such as low water salinity, thus sharing the same habitat with *S. olivacea*. Unlike *S. olivacea*, *S. paramamosain* and *S. tranquebarica* are known to inhabit estuaries and river mouths with higher salinities (Ikhwanuddin et al., 2011; Keenan, Davie & Mann, 1998).

The invasion of *S. beauforti* onto the *Scylla* population in Marudu Bay is most likely originating from the first reported infection site—Sandakan Bay, approximately 170 km apart. Various vectors, human-mediated or non-human-mediated, are possible in the transfer of this parasite. Both bays are home to two towns that focus on fisheries and marine products, Kota Marudu and Sandakan. Traders often travel between these two towns and it is highly possible that infected mud crabs from Sandakan Bay were accidentally released or escaped into the wild. Another possible vector might be due to shipping, such as via ballast water and hull fouling. Sandakan, the former capital of British North Borneo before the formation of Malaysia, is an important international transportation port with heavy water traffic. Located northwest of Sandakan, larvae and adults of *S. beauforti* are easily transferred to Marudu Bay when ships pass through. Similar human-mediated dispersal vectors were postulated for the northward expansion of rhizocephalan *L. panopaei* in the northwest Atlantic (Freeman, Blakeslee & Fowler, 2013). Their expansion in the Chesapeake Bay changed the local mud crab species composition, causing a drastic drop in the population of the once-flourished xanthid, *Eurypanopeus depressus* (Smith, 1981) and made the non-susceptible rare xanthid species, *Dyspanopeus sayi* (Smith, 1981) the dominant mud crab species (Andrews, 1980). This is alarming as mud crab traders from Sarawak and peninsular Malaysia occasionally obtain live mud crabs from Sabah due to their lower price, higher quantity and larger size. If infected crabs were transported and accidentally released, there is a high chance that *S. beauforti* will become prevalent as *S. olivacea* is the most abundant *Scylla* species in Sarawak and peninsular Malaysia (Ikhwanuddin et al., 2011; Waiho, Fazhan & Ikhwanuddin, 2016; Waiho et al., 2017). This epidemic might even cross the border and reach southern Thailand via land route, where *S. olivacea* is also abundantly found (Moser et al., 2005).

In summary, the present study reports the occurrence of six distinct types of crabs with abnormalities in the *S. olivacea* population of Marudu Bay, Sabah, Malaysia. This matter is crucial as it involves a commercially important, edible crab species and that locals showed a higher preference for these abnormal crabs over normal crabs. Future in depth description of the life cycle and characteristics of rhizocephalan parasite, *S. beauforti* are recommended in order to determine the edible status of *S. olivacea*. In addition, the prevalence of *S. beauforti* infestation on the *S. olivacea* population could be reduced if parasitized crabs are harvested regardless of size and maturation status as they do not contribute to the population recruitment, but instead are competitors with healthy individuals for resources and are sources for the spreading of infectious *S. beauforti* larvae.

##  Supplemental Information

10.7717/peerj.3419/supp-1 Table S1 Partial COI sequences of Sacculinids species retrieved from Genbank and their corresponding accession numbers.

10.7717/peerj.3419/supp-2 Data S1 Supplementary data S1

## Additional Information and Declarations

### DNA Deposition

The following information was supplied regarding the deposition of DNA sequences: The partial COI sequence of *Sacculina beauforti* described here is accessible via GenBank accession number KX426583.

### Data Availability

The following information was supplied regarding data availability: The raw data has been supplied as a Supplementary File.
