# Supplementary material for: Infestation of parasitic rhizocephalan barnacles Sacculina beauforti (Cirripedia, Rhizocephala) in edible mud crab, Scylla olivacea
Source: PeerJ. 2017 Jun 30;5:e3419. doi: 10.7717/peerj.3419 (PMC5494170; doi:10.7717/peerj.3419)
Supplement: Table S1 [file peerj-05-3419-s001.docx]

| **Species** | **Accession numbers** |
| --- | --- |
| *Sacculina carcini 2* | DQ059773.1 |
| *Sacculina leptodiae* | AY265376.1 |
| *Sacculina carcini 1* | AY117692.1 |
| *Ommatogaster nana* | AB602398.1 |
| *Peltogaster paguri* | KT209076.1 |
| *Sacculina yatsui* | AB197808.1 |
| *Sacculina yatsui* | AB197809.1 |
| *Sacculina imberbis* | AB197806.1 |
| *Sacculina sp. E-NZ* | EF521402.1 |
| *Sacculina granifera* | DQ059779.1 |
| *Sacculina leptodiae* | AY265376.1 |
| *Sacculina sinensis* | AY265377.1 |
| *Sacculina oblonga* | AY265375.1 |
| *Sacculina confragosa* | AY265374.1 |
| *Heterosaccus lunatus* | DQ059778.1 |
| *Sacculina beauforti* | KX426583 (current study) |
| *Loxothylacus panopaei 1* | KF530194.1 |
| *Loxothylacus panopaei 2* | HQ848070.1 |
| *Heterosaccus dollfusi* | AY117691.1 |
